# A High-Precision Multi-Beam Optical Measurement Method for Cylindrical Surface Profile

**DOI:** 10.3390/mi14081555

**Published:** 2023-08-03

**Authors:** Yinghong Zhou, Zhiliang Wu, Nian Cai, Daohua Zhan, Shaoqiu Xu, Meiyun Chen, Guang Zhou, Han Wang

**Affiliations:** 1 School of Information Engineering, Guangdong University of Technology, Guangzhou 510006, China; 2 School of Electromechanical Engineering, Guangdong University of Technology, Guangzhou 510006, China; 3 School of Automation, Guangdong University of Technology, Guangzhou 510006, China; chenmeiyun2017@163.com

**Keywords:** multi-beam optical measurement, cylindrical surface profile, machine vision, surface normal vector

## Abstract

To automatically measure the surface profile of a cylindrical workpiece, a high-precision multi-beam optical method is proposed in this paper. First, some successive images for the cylindrical workpiece’s surface are acquired by a multi-beam angle sensor under different light directions. Then, the light directions are estimated based on the feature regions in the images to calculate surface normal vectors. Finally, according to the relationship of the surface normal vector and the vertical section of the workpiece’s surface, a depth map is reconstructed to achieve the curvature surface, which can be employed to measure the curvature radius of the cylindrical workpiece’s surface. Experimental results indicate that the proposed measurement method can achieve good measurement precision with a mean error of the curvature radius of a workpiece’s surface of 0.89% at a reasonable speed of 10.226 s, which is superior to some existing methods.

## 1. Introduction

Ultra-precision industrial components have been widely used in aerospace, national defense, biomedicine, communication, microelectronics and other high-tech fields [1,2,3,4,5,6,7], which require high-precision surface profiles. Accurate measurement of curved surface profiles is beneficial for the quality check (QC) of cylindrical components and further influences the reliability of the equipment involving these cylindrical components. Also, it can play an important role in improving machining machines.

Two types of measurement methods are commonly used to accurately measure the surface profile of industrial components, which are contact measurement [8] and non-contact measurement [9]. Contact measurement devices, such as coordinate measuring machines [8], have the advantages of high precision, convenient operation and strong versatility. However, they are time-consuming for surface profile inspection and vulnerable to environmental factors such as temperature. Moreover, their contact probes can possibly cause damage to the surface of the industrial component and wear on themselves. Non-contact measurement devices involve industrial CT [9] and technologies including laser triangulation [10], structured-light vision technology [11,12], binocular stereo vision technology [13] and photometric stereo vision technology [14,15,16,17,18,19]. They have the advantages of high efficiency, convenient operation, high degree of automation, no need for probe radius compensation and non-contact measurement of ultra-precision workpieces. So, they have been more and more frequently employed for the surface profile inspection of industrial components. However, most of them are easily affected by the reflection characteristics of industrial components.

To improve the efficiency and precision of surface profile measurement for cylindrical industrial components, a high-precision multi-beam optical detection method is proposed for the fast and non-contact measurement of the cylindrical surface profile of industrial components. First, our previously designed non-contact measurement device [20] is used to acquire the surface images of the cylindrical industrial component under different light directions. The acquired images are superimposed to calculate the light direction. The surface normal vector of the measured component is calculated and separately analyzed in three dimensions. Depth information is derived based on the fitted surface normal vector. Finally, by means of the depth information of the cylinder, the curvature circle of the cylinder is calculated with the least-squares method. Thus, the curvature radius of the cylindrical industrial component is calculated using the curvature circle.

## 2. Non-Contact Measurement Device

**Figure 1:**
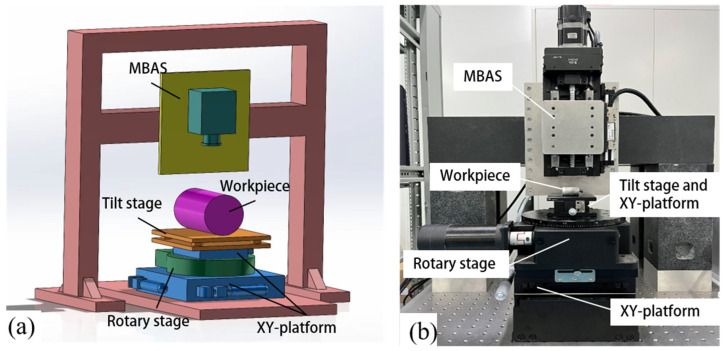
Non-contact measurement device for high-precision cylindrical surface multi-beam optical inspection: (**a**) design of the device, (**b**) experimental device.

All the images for the subsequent surface profile measurement were acquired by our previously designed non-contact measurement device, which was designed for cylindrical surface multi-beam optical inspection [20]. As illustrated in Figure 1, the device consists of two key subsystems, which are the multi-beam angle sensor (MBAS) and the rotating subsystem.

**Figure 2:**
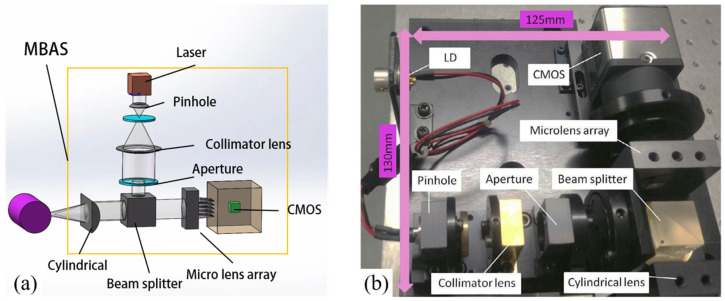
Architecture of the MBAS: (**a**) design of MBAS, (**b**) experimental MBAS.

Figure 2 illustrates the architecture of the MBAS. The laser beam passing through the pinhole is first collimated by the collimator lens, and then bent by the beam splitter and projected onto the surface of the measured workpiece through the cylindrical lens. The cylindrical lens is used to eliminate the effects of cylindrical curvature. After passing through the cylindrical lens and the beam splitter, the reflected beam is split and focused into multiple sub-beams by the microlens array. A CMOS camera with 1920 × 2560 resolution is mounted along the vertical axis to observe the sub-beams.

The rotating subsystem involves a rotary stage, two XY platforms and a tilt stage. The rotary stage is mounted between two XY platforms. When MBAS is used to measure cylindrical surfaces, the tilt table is fixed. The workpiece mounted on a tilt stage is rotated by the rotary stage and its top-view images at different rotation angles are collected for surface curvature measurement.

The spindle of the rotary stage is the most important part in the assembly of any curvature measurement device. The Z-axis is the main rotation axis of the image acquisition system and should be aligned with the axis of the rotary stage to ensure that they are collinear. This alignment can be achieved by adjusting the position of the two XY platforms. The upper XY platform is used to minimize the spindle error between the workpiece and the turntable, while the lower XY platform is used to approach the perfect position between the multi-beam sensor and the turntable. After elaborate adjustment, the workpiece, the cylindrical lens and the CMOS are colinear, which can avoid the workpiece moving out of the imaging range of the CMOS during its rotation.

**Figure 3:**
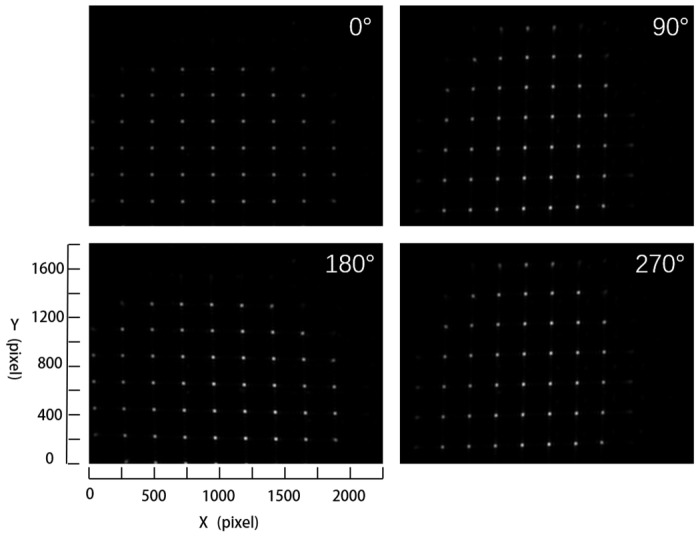
Images acquired using the measurement device under different light directions.

When the workpiece is wholly rotated, several images of the workpiece at different rotation angles, i.e., under different light directions, are acquired by the experimental measurement device, as shown in Figure 3. The white spots visible in the images are due to the focused sub-beams. It is noted that each white spot can be considered as a feature region for our proposed measurement method. The direction of the light source can be estimated using the feature regions and then the surface normal vector of the workpiece is calculated with Lambert’s model. The cylindrical surface of the workpiece is reconstructed using the surface normal vector. Thus, the surface curvature of the workpiece can be calculated using the reconstructed cylindrical surface. Detailed explanations are provided in Section 3.

## 3. Proposed Measurement Methodology

**Figure 4:**
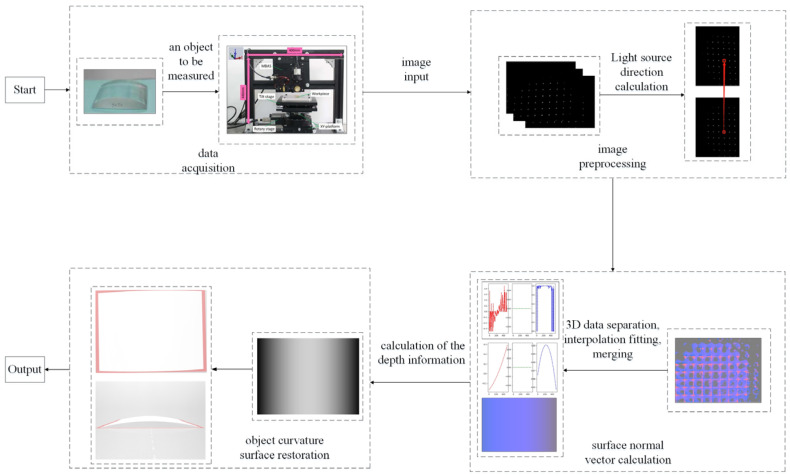
Flowchart of the proposed multi-beam optical measurement method.

The multi-beam optical surface measurement method is proposed for cylindrical surface profile based on the optics theory, which is sketched in Figure 4. First, the relationship between the object surface normal direction, the light direction and the image brightness is determined based on the reflection model of the object surface. Then, multiple top-view images under different light directions are collected for the calculation of surface normal vector. Next, depth information is inferred based on the least-squares method (LSM). Finally, the curvature circle in the Z direction is reconstructed from the projection shape of depth information in the cylindrical axis direction, which is used to calculate the curvature radius of the cylindrical workpiece.

### 3.1. Calculation of Light Direction

The surface normal vector is necessary for depth image reconstruction of the cylindrical workpiece to calculate its curvature radius, which can be calculated by combining the light direction and the intensities of feature regions. Since the workpiece is rotating via the rotary stage, the beam is reflected in different directions due to the curved surface of the workpiece.

**Figure 5:**
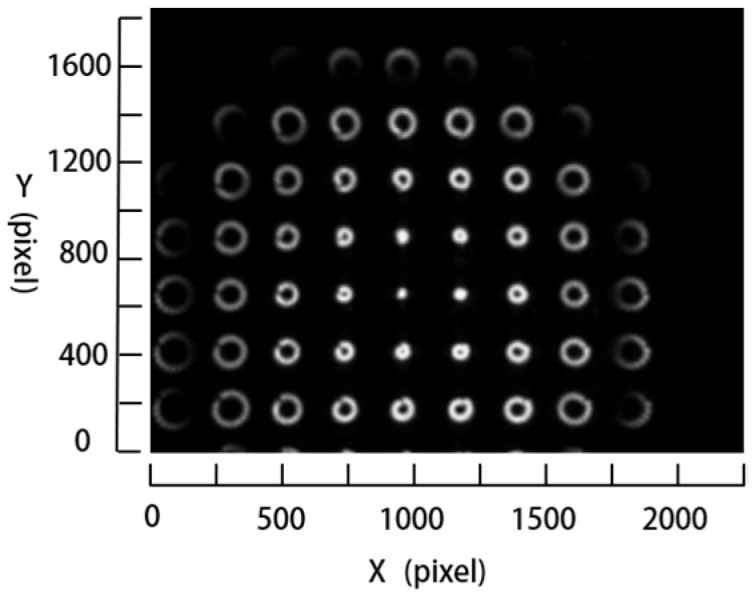
Movement traces of feature regions.

Thus, after the reflected light passes through the beam splitter and the microlens array, the image appears bright to dark from the middle to the periphery of the cylindrical workpiece. Thus, the change in the rotation direction can be estimated from the change in the movement direction of feature regions in the image. As shown in Figure 5, the movement traces of feature regions can be observed after superimposing binarized images under different light directions achieved by the adaptive thresholding method [21]. The edges of the cylindrical workpiece reflect light out of the camera’s field of view, and the surrounding feature regions are blurred, so the central group of feature regions is selected and used for the rotation direction estimation. The calculation of light direction is described below.

For a grayscale image f(x,y) with a size of M×N pixels, its p+q order geometric moment $m_{pq}$ is expressed as

(1) $$m_{pq}=\sum_{x=1}^{M}\sum_{y=1}^{N}x^{p}y^{q}f\left(x,y\right)$$

where p,q∈{0,1}. The center of gravity $\left(x_{c},y_{c}\right)$ is expressed as

(2) $$x_{c}=\frac{m_{10}}{m_{00}},y_{c}=\frac{m_{01}}{m_{00}}$$

**Figure 6:**
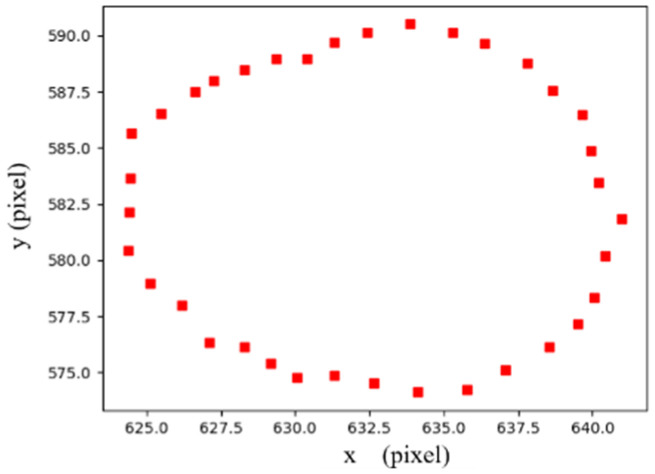
The gravity centers of the feature regions under different light directions.

All the centers of gravity of the feature regions in the central group are calculated with (2), which form a circle-like appearance, as shown in Figure 6. Thus, the angles between adjacent centers can be determined based on the minimal enclosing circle. The light direction L can be represented as

(3) L=[cos θ,0,μsin θ]

where θ is the angle between adjacent centers and μ is a constant related to the height difference between the camera and the platform.

### 3.2. Calculation of Surface Normal Vector

The relationship between the light direction, the surface normal vector of the object and the corresponding acquired image is expressed as

(4) $$I=\rho L^{T}N$$

where I is the pixel brightness value, ρ is the reflection coefficient of the object surface, L is the unit direction vector of illumination and N is the unit normal vector of the object surface.

Due to the splitting of the reflected light beam, the surface normal vector information collected from the camera is discrete and noisy. If it is directly used for depth image reconstruction, an uneven surface will be reconstructed, which is not consistent with the physical surface of the cylindrical workpiece.

In order to achieve continuous surface normal vector information, polynomial fitting is performed on the *x*, *y* and *z* components of the surface normal vector, which is formulated as

(5) $$P_{n}\left(y_{i}\right)=a_{0}+a_{1}x_{i}+a_{2}{x_{i}}^{2}+\cdots +a_{n-1}{x_{i}}^{n-1}+a_{n}{x_{i}}^{n}$$

where $a_{0},a_{1},a_{2},\cdots ,a_{n-1},a_{n}$ are the polynomial coefficients.

**Figure 7:**
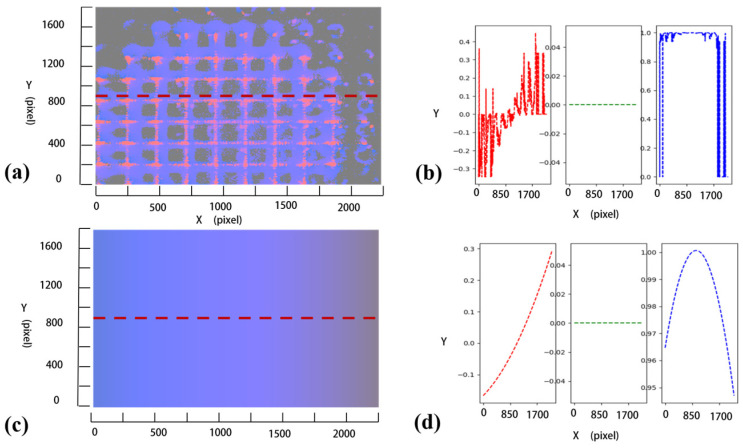
Surface normal vector map with/without polynomial fitting: (**a**) surface normal vector map without polynomial fitting, (**b**) individual components of the surface normal vectors with respect to the line in (**a**) (from left to right, they correspond to the X-, Y-, and Z-axis, respectively), (**c**) surface normal vector map with polynomial fitting, (**d**) individual components of the surface normal vectors with respect to the line in (**c**) (from left to right, they correspond to the X-, Y-, and Z-axis, respectively).

An example of a surface normal vector with/without polynomial fitting is illustrated in Figure 7. As shown in Figure 7a,c, the uneven surface normal vector map becomes smooth after polynomial fitting. Individual components of surface normal vector in the same row are displayed in Figure 7b and Figure 7d, respectively, as illustrated in Figure 7a,c.

### 3.3. Depth Map Reconstruction and Calculation of Curvature Radius

Assume that a surface pixel (*i*, *j*) and its 4 neighbors are on the same tangent plane. The vector corresponding to the right neighbor pixel (*i* + 1, *j*) is

(6) $$\left(i+1,j,z_{i+1,j}\right)-\left(i,j,z_{i,j}\right)=\left(1,0,z_{i+1,j}-z_{i,j}\right)$$

where *z* (*i*, *j*) represents the depth information of the pixel (*i*, *j*). This vector is perpendicular to the surface normal vector $N_{i,j}$ of the point, so

(7) $$\left(1,0,z_{i+1,j}-z_{i,j}\right)\cdot N_{i,j}=0$$

(8) $$z_{i+1,j}-z_{i,j}=-\frac{N_{i,j}^{x}}{N_{i,j}^{z}}$$

In the same way, for each pixel (*i*, *j*), we can obtain the equation set in four directions as

(9) $$\left[\begin{array}{c} 1\;\;0-1\;\;0\;\;0\\ 0\;\;1-1\;\;0\;\;0\\ 0\;\;0-1\;\;1\;\;0\\ 0\;\;0-1\;\;0\;1 \end{array}\right]\left[\begin{array}{c} z_{i-1,j}\\ z_{i,j-1}\\ z_{i,j}\\ z_{i+1,j}\\ z_{i,j+1} \end{array}\right]=\left[\begin{array}{c} N_{i,j}^{x}/N_{i,j}^{z}\\ N_{i,j}^{y}/N_{i,j}^{z}\\ -N_{i,j}^{x}/N_{i,j}^{z}\\ -N_{i,j}^{y}/N_{i,j}^{z} \end{array}\right]$$

which can be written as a linear system of equations with respect to *z*, formulated as

(10) Mz=v

Then, *z* can be solved using

(11) $$z={\left(M^{T}M\right)}^{-1}M^{T}v$$

By means of (11), the depth image is reconstructed for the surface of the cylindrical workpiece. A curvature circle in the *z* direction can be determined from the projection shape of depth information in the cylindrical axis direction via the least-square method, which can be utilized to calculate the curvature radius of the curved surface of the cylindrical workpiece.

## 4. Experimental Results and Discussions

All experiments were conducted on a computer with Intel Xeon E5-2630 v3 2.40 GHz CPU, Santa Clara, CA, USA and Nvidia Quadro M400 8 GB GPU, Santa Clara, CA, USA. And the diameter of the cylindrical workpiece used for the experiment is 200 mm. Starting from the initial position, MBAS took one picture for every 10 degrees of rotation of the rotating platform, for a total of 36 images. We used two metrics to objectively compare our method with other methods, which are the mean error of the curvature radius and running time. The mean error of the curvature radius was obtained by counting the curvature errors of the corresponding workpiece surfaces in the XY plane.

To validate the effect of different fitting methods on the measurement result, we compare the unfitted method, the cubic spline interpolation (CSI) and the polynomial fitting method (PF). As shown in Table 1, since the surface normal vector information collected by MBAS is discrete and noisy, the radius of the curvature measurement results obtained using the unfitted normal vectors have large errors. CSI improves the dispersion problem but does not solve the noise problem, so there is still some error. The PF achieves the best measurement accuracy among these three with the a priori knowledge that the cylindrical cross section can be fitted with a quadratic function.

To validate the influence of solving surface normal vector on the proposed measurement method, we compared the least-square method with L1 residual minimization (L1) [22] and sparse Bayesian learning (SBL) [23]. As indicated in Table 2, since SBL is a probabilistic-model-based approach for optimizing objective functions, which are very expensive or slow to evaluate, the key idea of this approach is to limit the evaluation phase of the objective function by spending more time choosing the next set of hyper-parameter values to try [24], resulting in severe underfitting in the case of small dataset sizes or large models [25]. The measurement method with SBL achieves the worst measurement performance with a mean error of the curvature radius of 15.71%. Due to the addition of an a priori constraint [26], the measurement method with L1 norm achieves much better measurement precision than that with SBL. Comparatively, the proposed measurement method with LS can achieve the best measurement precision, with a 0.89% mean radius of curvature error at the fastest speed of 10.266 s. This is because LS is easily implemented and solved [27]. Also, LS prefers to learn features with small weights, which means that it can capture as much information as possible [28] to improve the reconstruction performance of the depth map.

**Figure 8:**
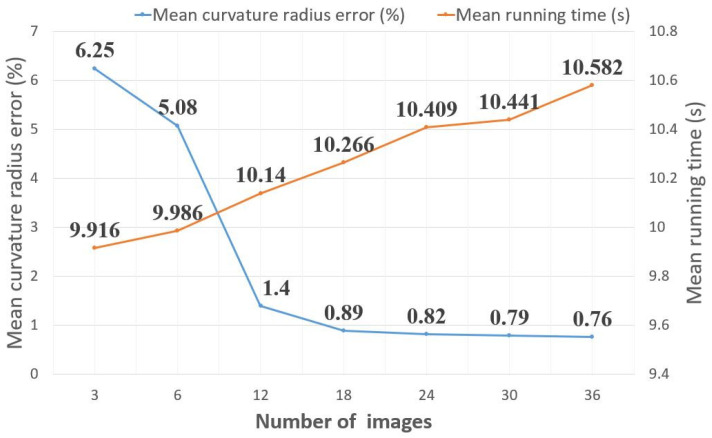
Mean curvature radius error and average running time of images with different light source directions.

Since the surface normal vector has three directions, at least three acquired images should be utilized to reconstruct the depth map of the cylindrical workpiece’s surface. To reveal the influence of the number of acquired images on the measurement performance, we performed the proposed measurement method with different numbers of acquired images, as illustrated in Figure 8. It can be seen that more images lead to lower error but more running time. This is because more spatial information can be extracted from more images. When more than 18 images are utilized for depth map reconstruction, the measurement precision improves slightly but with relatively more running time with the increase in the number of images. So, in our work, 18 acquired images were used for the proposed measurement method.

To validate our proposed measurement method, we compared it with the state-of-the-art measurement work in [20], which is an angle difference calculation (ADC) method. Since our proposed measurement method is similar to the photometric stereo method, we also compared it with several photometric stereo methods [29,30]. 

**Figure 9:**
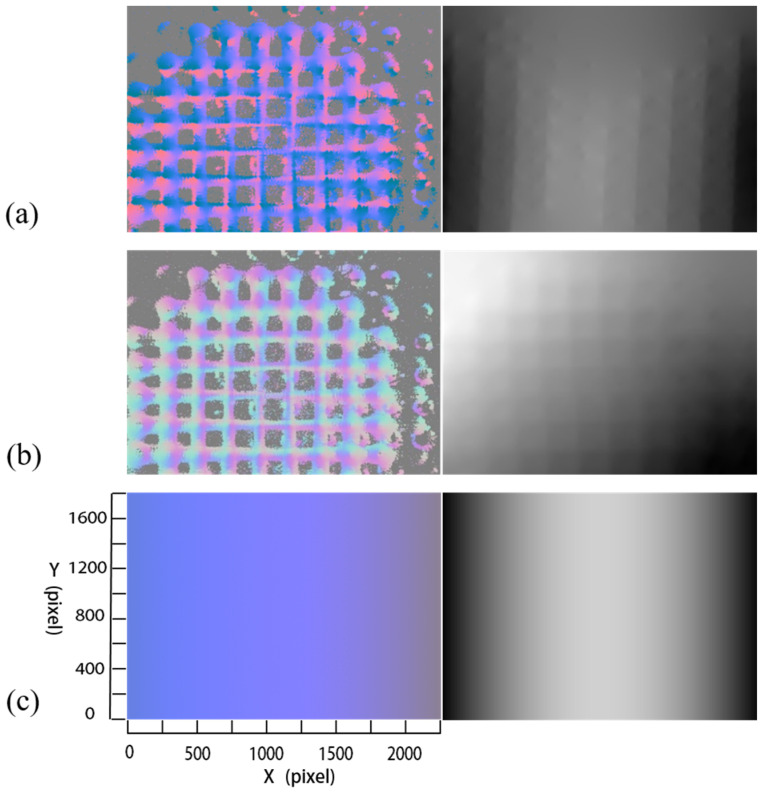
Surface normal maps and corresponding depth maps achieved using (**a**) the method in [29]; (**b**) the method in [30]; (**c**) ours.

As shown in Figure 9, due to the splitting of the reflected light beam, the information collected from the camera for calculating the surface normal vector is discrete and even noisy. Thus, the surface normal maps achieved through the methods in [29,30] appear to be rough, which means that many dimple patterns emerge in their reconstructed depth maps of the workpiece’s surface. The facts can explain well why the two methods perform very bad measurements in terms of a more than 50% mean error of the curvature radius, as shown in Table 3. It is noted that the method in [30] takes more time for measurement by a factor of more than 22 compared with the method in [29] and ours. This is because it formulates the joint recovery of reflectance, lighting and geometry as a variational problem solved by a “discretize-then-optimize” approach, which is greatly time-consuming.

Comparatively, due to the polynomial fitting of a normal vector based on prior knowledge and a simple scheme of depth map reconstruction, our proposed measurement method achieves a smooth surface normal map to reconstruct a smooth depth map. So, our method achieves the best measurement precision among all the methods at a reasonable speed of 10.266 s. In particular, it utilizes an automatic calculation method to achieve better measurement precision than the ADC, which is a promising but manual measurement method, with mean errors of curvature radius of 0.89% vs. 1.00%. The facts indicate the feasibility of our proposed measurement method in real measurements.

## 5. Conclusions

In this paper, a high-precision multi-beam optics method is proposed to measure the surface profile of a cylindrical workpiece. First, we introduce a non-contact measurement device to acquire the images under different light directions. Then, the light direction is estimated according to the movement trace of the gravity centers of image feature regions. Next, the depth map of the cylindrical workpiece’s surface is derived after performing polynomial fitting on the surface normal vector components. Finally, the curvature radius for the curve surface of the cylindrical workpiece is calculated by the depth map. The experimental results indicate that the least-squares scheme is appropriate for solving the surface normal vector, which is a significant step of the proposed measurement method. The comparison experiments indicate that the proposed measurement method achieves better measurement precision for the cylindrical workpiece’s surface profile at a reasonable speed compared with other existing methods. Specifically, it measures the mean error of the curvature radius of a workpiece’s surface as 0.89% at the speed of 10.226 s.

## Figures and Tables

**Table 1:** Effect of different fitting methods on measurement results.

Method	Unfitted	CSI	PF
Mean error of curvature radius (%)	>50	10.04	0.89

**Table 2:** Measurement results via the proposed methods with different schemes for surface normal vector.

Method	L1 [22]	SBL [23]	Ours
Mean error of curvature radius (%)	1.23	15.71	0.89
Average running time (s)	206.897	976.638	10.266

**Table 3:** Measurement of cylindrical surface profile via different methods.

Method	PS [29]	UPS [30]	ADC [20]	Ours
Calculation methods	Automatic	Automatic	Manual	Automatic
Mean error of curvature radius (%)	>50.00	>50.00	1.00	0.89
Average running time (s)	9.147	230.854	×	10.266

## Data Availability

The data that support the finding of this study are available from the corresponding author upon reasonable request.
